# Maternal health care seeking by rural Tibetan women: characteristics of women delivering at a newly-constructed birth center in western China

**DOI:** 10.1186/s12884-015-0634-9

**Published:** 2015-09-22

**Authors:** Kunchok Gyaltsen, Jessica D. Gipson, Lhusham Gyal, Tsering Kyi, Andrew L. Hicks, Anne R. Pebley

**Affiliations:** Tso-ngon (Qinghai) University Tibetan Medical College, No. 16 Kunlun Road., Xining City, Qinghai Province 81001 P.R. China; Department of Community Health Sciences, UCLA Fielding School of Public Health, 650 Charles E. Young Drive South CHS 46-071B, Los Angeles, CA 90095-1772 USA; Tibetan Birth and Training Center, Tongren County of Huannan Prefecture, Qinghai, P.R. China; CCPR Statistics and Methods Core, California Center for Population Research, University of California Los Angeles, Los Angeles, CA USA; Current address: Department of Health Care Policy, Harvard Medical School, Boston, MA USA; California Center for Population Research, University of California Los Angeles, Los Angeles, CA USA

## Abstract

**Background:**

Increasing skilled birth attendance at delivery is key to reducing maternal mortality, particularly among marginalized populations. Despite China’s successful rollout of a national policy to promote facility deliveries, challenges remain among rural and ethnic minority populations. In response, a Tibetan Birth and Training Center (TBTC) was constructed in 2010 to provide high-quality obstetric care in a home-like environment to a predominantly Tibetan population in Tso-ngon (Qinghai) province in western China to improve maternal care in the region. This study examines if and how first users of the TBTC differ from women in the broader community, and how this information may inform subsequent maternal health care interventions in this area.

**Methods:**

Trained, Tibetan interviewers administered a face-to-face, quantitative questionnaire to two groups of married, Tibetan women: women who had delivered at the TBTC between June 2011-June 2012 (*n* = 114) and a non-equivalent comparison group of women from the same communities who had delivered in the last two years, but not at the TBTC (*n* = 108). Chi-squared and ANOVA tests were conducted to detect differences between the samples.

**Results:**

There were no significant differences between the samples in education or income; however, women from the TBTC sample were significantly younger (25.55 vs. 28.16 years; *p* < 0.001) and had fewer children (1.54 vs. 1.70; *p* = 0.05). Items measuring maternity health care-seeking and perceived importance of health facility amenities indicated minimal differences between the samples. However, as compared to the community sample, the TBTC sample had a greater proportion of women who reported having the final say regarding where to deliver (26 % vs. 14 %; *p* = 0.02) and having a friend or family member who delivered at home (50 % vs. 28 %; *p* < 0.001).

**Conclusions:**

Findings did not support the hypothesis that the TBTC attracts lower-income, less-educated women. Minimal differences in women's characteristics and perceptions regarding delivery care between the two samples suggest that the TBTC is serving a broad cross-section of women. Differences between the samples with respect to delivery care decision-making and desire for skilled birth care underscore areas that may be further explored and supported in subsequent efforts to promote facility delivery in this population, and similar populations, of women.

## Background

Globally, an estimated 800 women die every day from preventable causes related to pregnancy and childbearing-related causes [1]. Key intervention strategies to prevent maternal deaths are focused on increasing the proportion of deliveries at health care facilities and the proportion attended by a skilled birth attendant [2, 3]. These interventions are particularly relevant for populations at increased risk of maternal mortality and in addressing unique cultural factors that may affect women’s use of services, e.g., rural, impoverished, and ethnic minority women [1, 4–6].

China has made considerable progress in reducing maternal mortality [7]. This progress, however, has been less consistent in rural areas of western China and areas inhabited by a higher proportion of ethnic minority populations. In 2010, the maternal mortality ratio (MMR) was 46.1 per 100,000 live births in western China, as compared to 29.1 for the central region and 17.8 for the eastern region [8–11]. Stark disparities in maternal mortality rates have persisted despite the implementation of a national policy promoting hospital-based childbirth and subsidization of maternal health care services through the New Cooperative Medical Scheme [12, 13]. Although recent evidence suggests that the urban–rural divide in maternal mortality may be narrowing [14], a recent review of provincial maternal mortality surveillance systems in China found that remote areas (i.e., Qinghai and Gansu provinces) had, on average, MMRs that were six- to nine-fold higher than the comparison region of Shanghai (Relative risks: 6.71–9.36), and that these disparities have not narrowed since 1999 [12].

In Qinghai province, a predominantly rural province in western China and the focus of this study, MMRs are estimated to be even higher than the western regional average, with an MMR of 128 in 1999 and 107 from 2000 to 2008 [12, 15, 16]. Although studies are limited from this area, a small community-based investigation (*n* = 279) conducted in 2003 among Tibetan women in Qinghai province found that only 30 % of women visited a health care provider at any time during their pregnancy and 93 % gave birth at home without a trained birth attendant [17]. A subsequent study (2004) conducted among Tibetan women living in another part of Qinghai province (*n* = 402) found that 52 % of women sought preventive care during pregnancy and 99 % gave birth without a trained birth attendant [18].

Few studies investigate the reasons for these persistent disparities in maternal mortality and maternal health care utilization among Tibetan populations [12, 18–20], and even more limited are interventions that integrate culturally-tailored aspects to improve maternity care utilization [6, 20–22]. Among the studies that have been conducted, the identified barriers are similar to those experienced in other settings [5] and are often framed within the Three Delays Model [23]: the delay in deciding to seek care, the delay in identifying and reaching a medical facility, and the delay in receiving adequate and appropriate treatment. In the context of western China and Tibet, barriers to care include limited access to trained birth attendants due to distance, difficult or limited transport, and additional costs not covered by government subsidies [17, 19]. Moreover, hospitals typically lack accommodations for family members and patients must pay for the additional expenses of travel and food. Anthropological evidence from these regions also indicate that language and cultural barriers may impede the use of Chinese medical facilities by Tibetan women [20]. One example is the way in which pregnancy and childbirth are viewed; most Tibetan women view pregnancy and childbirth as a normal process and one that does not require going to a hospital [20].

To address maternal mortality among Tibetan women in Tso-ngon (Qinghai)^1^ province, a team of Tibetan doctors and public health practitioners worked with the Tibetan Healing Fund and local officials to construct the Tibetan Birth and Training Center (TBTC) in 2010 [19, 24]. The goals of the Center are two-fold: 1) to improve maternity care in in the local region, and 2) to develop and refine a new model for improved maternity care in Tibetan (and other hard-to-reach rural) areas of China. In addition to community health worker recruitment and training and peer education programs, the TBTC provides linguistically and culturally-appropriate maternity services for the predominantly Tibetan population in Rongwo, Rebkong (Tongren) County, Malho (Huangnan) Tibetan Autonomous Prefecture [19].

The TBTC is based on a birthing center model and aims to attract Tibetan and other women who would not normally deliver in a hospital, by providing high-quality obstetric/gynecologic care in a home-like environment. Women and their families stay in suites equipped with traditional Tibetan heated beds (*kang*), cooking facilities, and bathrooms with showers. Traditional Tibetan childbirth practices are also integrated into the services as desired by the women and their families [19].

### Aim

Building on findings from the larger study documenting the components of the TBTC and the related efforts to address maternal mortality in this area [19], as well as a description of the motivations for and experiences of women who delivered at the TBTC [25], in this paper we sought to explore the potential selectivity of women who deliver in this new facility. To achieve this aim, we investigate and compare the sociodemographic and maternity health care-seeking characteristics of two samples of women: women who delivered at the TBTC and women from the same communities who delivered elsewhere (i.e., at home and/or in a health facility).

While the purpose of the paper is primarily descriptive, we are guided by several hypotheses about which women are more likely to be attracted to a novel facility such as the TBTC. The TBTC is novel for pregnant women in the area because it is neither a standard government-run hospital, nor is it home delivery. Based on the Diffusion of Innovation theory [26], the Health Belief Model [27], and the Theory of Planned Behavior [28], we might expect that women selecting this novel option would be younger, more educated, have a higher perceived risk of childbirth complications, and express more favorable attitudes to maternity health care-seeking. On the other hand, the TBTC was designed, at least in part, to attract underserved rural women who would be less comfortable in a hospital setting, but who prefer (or whose families prefer) skilled delivery care. This perspective suggests that TBTC users might be less educated and from lower income families. The issue is particularly important in the Tibetan Plateau and in other poor regions because programs like TBTC are intended to increase the use of skilled delivery attendance rather than merely to replace existing hospital based care. In this paper, we will assess which types of women deliver at TBTC compared to the general population to attempt to address these questions.

## Methods

### Setting

Our study was conducted in Rebkong (Tongren) County in Tso-ngon (Qinghai) Province in P.R. China, 170 km southeast of Xining, the capital city. Tso-ngon (Qinghai) Province is in western China on the Tibetan plateau and is part of the traditional Tibetan region of Amdo. Although Qinghai Province has experienced rapid growth and urbanization over the past decade – from a 2003 GDP of US$1814 urban and $483 rural [18] to a combined 2013 GDP (urban and rural) of $3540 [29] – Qinghai remains one of the poorest provinces in China. Rebkong (Tongren) County is located in Malho (Huangnan) Tibetan Autonomous Prefecture, one of five such prefectures in Qinghai. The vast majority of Rebkong residents are Tibetan, with other residents being Han, Hui, or members of other ethnic groups. Most Tibetans in this area are farmers or nomadic herders; however, the area is also known for bauxite mining (for aluminum).

Available health care facilities include small, village clinics attended by village doctors (typically with little or no obstetric training), and County and Provincial hospitals predominantly staffed by Han Chinese physicians and nurses [19]. In Tibetan areas, there are also a number of Tibetan medicine hospitals – as well as Chinese (traditional) medicine hospitals. Tibetan medicine hospitals do not provide maternity care and there are few trained birth attendants available outside of County and Provincial hospitals. Access to health care for Tibetan populations is further complicated by long and difficult travel, costs for the stay and medicine, lack of hospital accommodations for accompanying family members, and linguistic and cultural barriers between Tibetan women and non-Tibetan health care providers and facilities.

### Study design

Two non-equivalent samples of Tibetan women were selected for this study: a clinic-derived sample and a community-derived sample. For the clinic-derived sample, all married, Tibetan women who had delivered at the TBTC between June 2011 - June 2012 were eligible for selection. Since data collection occurred in September-October 2012, this timeframe was selected since it would capture women who would have recently delivered and also had time to recover from birth. Given local beliefs regarding the vulnerability of mothers and newborns, it was not appropriate to have non-family members making household visits soon after birth [20]. This clinic sample was selected in two stages. First, using a random number generator in Stata 13, women’s names were selected from the patient registers indicating all women who had delivered at the TBTC during this time period. The patient registers indicated that 619 women delivered during the study period. Women who lived in more remote and inaccessible locations were dropped from the sample, because of the difficulty of reaching these regions. In total, 114 women were selected from the clinic sample and agreed to take part in the survey.

The second group consisted of a non-equivalent comparison group of married, Tibetan women who had given birth in the last two years, but did not deliver at the TBTC. Since fertility is low in Rebkong County – estimated in 2000 as 1.2 births per woman – we expanded the reference period to two years for the community sample to facilitate recruitment of women who had recently delivered [30]. These women were selected by asking women from the clinic sample and local health workers for names of other women in their communities who had a delivery during the observation period.

### Data collection

The study employed a 45–60-minute, interviewer-administered questionnaire that incorporated elements of the Three Delays Model [23] the Theory of Planned Behavior [28], the Health Belief Model [27], and inputs from an earlier community-based study [17]. The survey was drafted and developed in English by the authors, then translated in to Tibetan, checked for comprehension, and further refined through pretesting by Tibetan team members. The survey instruments included sociodemographic information and pregnancy history, as well as measures to test theoretical constructs relevant to this investigation. For example, the notions of perceived susceptibility and normative beliefs as predictors of using the TBTC were measured through women’s reports of their friends/family members delivering at home and women’s reports of their friends/family members experiencing a pregnancy- or delivery-related complication [27, 28]. Lastly, perceptions of maternity health care and health care-seeking were included from items based on the Three Delays model and quality of care measures, including the perceived need for a skilled birth attendant, knowledge of where to go in case of obstetric emergency, and perceived ability to receive prompt care once at a facility [23]. These items were reported on a 1–5 scale, with 5 representing strong agreement. Women were also asked to report on the perceived importance of health care facility qualities and amenities (e.g., ability to communicate in Tibetan; room with a traditional Tibetan bed; having a female doctor, etc.). These items were measured on a 1–5 scale, with 5 representing ‘very important’.

Five Tibetan masters’ students specializing in medicine were trained in the study protocol and then assisted in the refinement and translation of the survey instrument after pretesting it in the study communities. Oral informed consent was obtained from each woman after the interviewer read a description of the project and answered any questions regarding the study. Women were provided a small, Tibetan-style scarf as compensation for their time. Approval to conduct the study was obtained from the UCLA Institutional Review Board. In total, 222 women were interviewed September – October 2012.

### Data analysis

The survey data were cleaned and verified by the research team. Data analyses were conducted in Stata 13.1. Measures from the community and clinic samples were compared using Chi-squared tests to detect differences in proportions and ANOVA tests to assess differences in means between the two samples. Significant differences are reported at the *p* ≤ 0.05 level.

## Results

Characteristics of the two study samples are provided in Table 1. Women from these samples were in their mid to late 20′s and had an average of 1–2 children. Just over one-half of women reported ever attending school, with husbands more likely to have ever attended school. The average yearly income level for the study population’s households was 21,502 RMB (US$3,542; median: 13,000 RMB).

**Table 1:** Characteristics of TBTC users and women in the community

Characteristic	Women who used TBC (*n* = 114)	Women in community (*n* = 108)	*P*-value^a^
Sociodemographic characteristics			
Age	25.55 (16–43)	28.16 (18–49)	<0.001
Number of children	1.54 (1–3)	1.7 (1–3)	0.05
Woman’s education			
Percentage of women who attended school	58.41	53.7	0.48
Of those who attended, average years of school	7.19 (0–26)	7.76 (0–26)	0.53
Husband’s age	27.95 (20–42)	30.05 (18–35)	0.00
Husband’s education			
Percentage of husband’s attending school	74.31	70.75	0.56
Of those attending, average years of school	7.34 (0–26)	7.45 (0–17)	0.88
Total yearly income (RMB)^b^	21,516 (~$3,421) (0–201,000)	21,487 (~$3,416) (0–120,000)	0.99
Percent living in two-generation household	78.94	68.52	0.08
Household assets/exposure to media (mean)			
Radio(s) in household	0.23 (0–2)	0.11 (0–1)	0.03
Television(s) in household	1.17 (0–4)	1.19 (0–3)	0.70
Computer(s) in household	0.13 (0–1)	0.22 (0–2)	0.09
Mobile phone(s) in household	2.46 (0–5)	2.47 (0–6)	0.92
Delivery experiences of friends and family			
Percent of women who reported a friend or family member delivering child at home	50.00	27.78	<0.001
Percent of women who reported a friend or family member having complications with childbirth	12.28	8.33	0.34
Delivery decision			
Woman herself had final say in where to deliver	26.32	13.89	0.02
Perceptions of maternity health care and health care-seeking^c^			
One does not need a doctor or nurse present during delivery/childbirth	1.48 (1–5)	1.93 (1–5)	0.01
I would not be treated well if I went to the prefecture hospital to deliver my baby	2.81 (1–5)	2.43 (1–5)	0.07
My family knows where to take me in case there is an emergency with my pregnancy or delivery	4.00 (1–5)	3.96 (1–5)	0.82
I would get care quickly if I went to the prefecture hospital for an emergency	3.75 (1–5)	3.86 (1–5)	0.54
I would be treated respectfully if I went to the prefecture hospital for pregnancy or delivery care	3.3 (1–5)	3.52 (1–5)	0.23
If I had an emergency with my pregnancy or delivery, I would be able to get to a healthcare facility quickly	2.54 (1–5)	2.51 (1–5)	0.91
I worry that if I have a complication with my pregnancy or delivery, my family will not know where to get help	2.63 (1–5)	2.38 (1–5)	0.25
It is better to deliver at home, than at the hospital	1.58 (1–5)	1.52 (1–5)	0.67
It is important to have a healthcare professional to assist with my delivery	4.79 (1–5)	4.78 (1–5)	0.85
Perceived importance of health care facility amenities^d^			
Facility is clean and comfortable	4.83 (1–5)	4.96 (3–5)	0.04
Ability to communicate to the doctor in Tibetan	4.88 (1–5)	4.88 (2–5)	0.99
Ability to communicate with the nurses in Tibetan	4.91 (2–5)	4.87 (2–5)	0.51
Ability to practice own beliefs during delivery	4.44 (1–5)	4.50 (2–5)	0.59
A private room during delivery	4.69 (1–5)	4.76 (2–5)	0.47
Access to own bathroom during delivery	4.83 (1–5)	4.70 (1–5)	0.16
Room with a traditional Tibetan bed (kang)	4.94 (2–5)	4.94 (2–5)	0.95
Ability for friends and family to stay in the room	4.79 (1–5)	4.75 (2–5)	0.64
Ability for family to cook during delivery	4.65 (1–5)	4.73 (2–5)	0.44
Ability to move around freely during delivery	4.52 (1–5)	4.45 (1–5)	0.51
Ability to communicate with doctor about the progress of the delivery	4.83 (2–5)	4.83 (2–5)	1.00
Having a female doctor	5.0 (5–5)	4.98 (3–5)	0.30

^a^ *P*-values report on differences based on chi-squared tests of proportions and anova tests of means

^b^Conversion October 2012 approximated 0.159 RMB = US$1

^c^On a scale of 1–5, with 5 indicating strong agreement

^d^Perceived importance on a scale of 1–5, with 5 indicating ‘very important’

Women and their husbands from the TBTC sample were younger (25.55 versus 28.16 years; *p* < 0.001), had fewer children (1.54 versus 1.70 children; *p* = 0.05), and were marginally more likely to live in an extended family household (79 % versus 68 %; *p* = 0.08), as compared to the community sample. There were no significant differences in husbands’ or wives’ educational levels or household income levels. Media exposure varied only for radio, with TBTC households being more likely to have a radio as compared to the community sample households (23 % of households versus 11 % of households; *p* = 0.03.

For the two measures on the reported delivery experiences of friends and family, only one measure was significantly different between the two groups. Half of the TBTC sample reported that a friend or family member had delivered a child at home, as compared to only 28 % of the community sample (*p* < 0.001). Although a greater proportion of the TBTC sample than the community sample reported that a friend or family member had childbirth complications (12 % versus 8 %), the difference was not statistically significant (*p* = 0.34).

Across the nine measures to assess perceptions of maternity health care and maternity health care-seeking, only two measures indicated significant differences between the two samples of women. Women in the TBTC sample were less likely to agree with the statement “One does not need a doctor or nurse present during delivery/childbirth”, as compared to women in the community sample (scores of 1.48 versus 1.93 on a 5-point scale, with 5 indicating strong agreement; *p* = 0.01). Women in the TBTC sample were marginally more likely to endorse the notion that “I would not be treated well if I went to the prefecture hospital to deliver my baby” (scores of 2.81 versus 2.43 on 5-point scale; *p* = 0.07).^2^

Lastly, women were asked 12 separate items about the perceived importance of specific health care facility amenities on a 5-point scale with 5 being “most important”. Across both samples, women reported high values for all items (4.45–5.0), with having a female doctor, being able to communicate with their doctor/nurse in Tibetan, having a room with a traditional Tibetan bed as the highest ranked items. There was a significant difference between the two groups for only one item – ‘facility is clean and comfortable’ – with women in the community sample rating this with higher importance as compared to the TBTC sample (4.96 vs. 4.83; *p* = 0.04).

## Discussion

China has demonstrated great success in reducing national-level maternal mortality rates [7]. Underlying this success, however, are large and persistent disparities in mortality rates across regions and populations [8–11]. The TBTC provides an innovative model to address these disparities by providing high-quality obstetric care to rural Tibetan women in a home-like environment, combined with an extensive outreach program [19]. This descriptive study of rural Tibetan women in Qinghai Province provides a comparison of early users of the TBTC and peers from their communities, as well as a test of hypotheses predicting characteristics of the early users of this newly-constructed birth center.

First, as hypothesized by theory underlying this study, we expected that the sample of TBTC users would be younger, more educated, have a higher perceived risk regarding childbirth complications, and express more favorable attitudes to maternity health care-seeking. Consistent with the hypotheses, women and their husbands in the TBTC sample were significantly younger than those in the community sample. The differences in education and levels of perceived risk, however, were not significant between the two groups, though they were in the hypothesized directions.

A second set of hypotheses posited that because the TBTC was designed to attract underserved rural women, these users are likely to be less educated and from lower income families. We did not, however, find any significant differences in education or income between the two samples. We also tested a measure of household wealth; this measure also did not differ between groups. Taken together, these findings generally indicate that the TBTC users and community sample of women were not significantly different, at least as could be detected in this study.

In comparing these findings to those from other settings [5], it is not surprising that we find mixed support for our competing hypotheses on the characteristics of women most likely to uptake maternity care services offered through the TBTC. As noted by Gabrysch and Campbell [5] in their review of the determinants of delivery care service use, factors influencing skilled delivery care vary greatly across sociocultural contexts and are influenced and confounded by a multitude of factors, including the level of development and available health care in the area. Similar to our findings, however, Gabrysch and Campbell find that in the vast majority of studies reviewed, younger women with lower parity births are more likely to have a skilled attendant at birth. As they note, younger women may place a higher value on pregnancy and seek support to ensure a successful outcome of the pregnancy [5].

An alternate interpretation of this finding, however, according to the Diffusion of Innovations theory is that younger women may be more likely to try the new model of care offered by the TBTC, a model that was previously unknown and unavailable to women prior to the 2010 opening of the TBTC [26]. An ‘early adopter’, according to the theory, has a more favorable attitude toward change and may have greater self-efficacy to enact this change [26]. In fact, women in the TBTC sample were more likely to have the final say in where they delivered, as compared to women in the community sample (26 % vs. 14 %; *p* = 0.02), lending further support for this mechanism. This finding deserves further exploration, however, since the majority of women in the sample reported that their mother or mother-in-law made the final decision as to where they would deliver [25]. Given evidence from other Asian settings citing the importance of husbands and extended family members, as well as women’s own decision-making power on maternal health care-seeking behavior [5, 31–33], further investigation is needed to determine how women and their family members are learning about the TBTC, and to what extent subsequent communication and intervention efforts should focus on women, as well as their extended family members, to promote skilled birth attendance at delivery.

Although there were nine items designed to assess potential delays or barriers to seeking delivery care, the majority of these items did not differ between the two samples. Women from the TBTC were more likely to agree with the items that they ‘would not be treated well’ if they went to the prefecture hospital, but were less likely to agree that ‘one does not need a doctor or nurse present during delivery/childbirth’. These findings, in combination with those indicating the high level of importance that women placed on being able to communicate with their physician in Tibetan and having more home-like amenities during delivery care suggest that the TBTC may be reaching underserved women who would not otherwise receive skilled delivery assistance from existing facilities. Further work is needed to determine the extent to which the TBTC may provide a viable option for women who prefer having a health care professional present, but for whom the existing health care facilities are not ideal.

Finally, although there are more recent efforts to describe and address the disparities in maternal mortality and morbidity within China (e.g., [8, 9]), in-depth examinations of specific populations, particularly rural and ethnic minority populations, are extremely limited. As such, further work is needed to understand and to attempt to address the unique health needs and persistent barriers to high-quality obstetric care among these populations, especially given the successes achieved nationally [34]. More broadly, there are few studies examining the characteristics and health-seeking behaviors of ethnic minority populations in China.

Three limitations should be noted in the interpretation of these findings. First, as a relatively small, exploratory study, the extent to which these findings can be generalized to the larger Tibetan population in this or any other area is limited. This study was not able to include data from women living in more remote areas due to logistic constraints and political unrest during the time of data collection; this omits information from nomadic women, for whom health care seeking is more challenging. Second, the small sample sizes may have limited the power to detect significant differences in some of theory-based measures designed to understand the motivations for using the TBTC (e.g., reporting of complications among friend or family member to assess risk perceptions). Last, given that the women in the community sample were referred by health workers or by women who had used the TBTC, they may have been more similar with respect to sociodemographic characteristics as a result of living and interacting in the same communities as compared to other women from these or other communities. Despite these limitations, however, this study provides a unique picture of a population of women and households from a remote location in Western China with unnecessarily high rates of maternal morbidity and mortality.

## Conclusion

In addition to providing descriptive and evaluative information on some of the first users of the TBTC, findings from this study also indicate directions in which subsequent research and programs could focus for the overall goal of reducing maternal mortality among Tibetan women living in rural, lower-income areas of western China. Specifically, the results suggest that the TBTC model itself, and some of its key features – such as providing services in women’s own language, combining high quality modern obstetrical care with Tibetan medicine practices and customs, and creating a home-like setting in which families who accompany women about to deliver have a place to stay – may be important strategies for reducing maternal mortality and morbidity in many areas of western China and similar regions.

## Abbreviations

TBTC: Tibetan birth and training center
UCLA: University of California, Los Angeles
RMB: Renminbi (Chinese currency)

## Footnotes

1 Locations in the Tibetan region often have both Tibetan and Chinese names. Throughout this article, we use Tibetan names followed by Chinese names in parentheses.

2 These items were also collapsed and tested using a principal components analysis; however, the findings did not differ from these simplified results.
